# Exploring teacher wellbeing in educational reforms: a Chinese perspective

**DOI:** 10.3389/fpsyg.2023.1265536

**Published:** 2023-11-10

**Authors:** Narentuya Ao, Sitong Zhang, Guoxiu Tian, Xiaoshuang Zhu, Xiaowei Kang

**Affiliations:** College of Teacher Education, Capital Normal University, Beijing, China

**Keywords:** teacher wellbeing, educational reforms, job characteristics, emotional regulation strategies, mindset

## Abstract

Teaching is a demanding profession and maintaining teacher wellbeing is significant in ensuring educational quality. However, teacher wellbeing is easily affected by educational reforms, and systematic research on this topic is still relatively rare. In China, with the enactment of the Double Reduction Policy in 2021, the job characteristics of primary and secondary school teachers have undergone various changes. Thus, the current study examined the new job characteristics that China’s Double Reduction Policy imposed on the wellbeing of school teachers and their relationships with teachers’ inner world (i.e., emotional regulation and mindset). A cross-sectional study was carried out from June to October 2022 across China, employing self-reporting questionnaires for data collection and analysis. With a random sample of 902 teachers, we investigated the associations between teacher wellbeing, job characteristics, emotional regulation strategies, and mindset. The results indicated that teachers showed a lower level of wellbeing after the educational reform. Higher job resources contributed positively to predicting teacher wellbeing, while higher job demands contributed negatively. Genuinely expressing had positive impacts on teacher wellbeing while surface acting had negative impacts and deep acting none. Mindset was found to affect emotional regulation strategies and teacher wellbeing simultaneously. These findings shed light on how teachers can appropriately regulate emotions and maintain wellbeing in the wake of educational reforms.

## Introduction

Teaching is a meaningful and influential profession (Collie et al., 2015) that influences the development of the youth of a nation. However, teaching is also demanding and challenging (Cook et al., 2016; Benevene et al., 2020), and long-term stress and heavy demands can be detrimental to teacher wellbeing (McGrath and Huntington, 2007). Teacher wellbeing refers to “teachers’ responses to the cognitive, emotional, health and social conditions pertaining to their work and their profession” (Viac and Fraser, 2020, p. 18). Low levels of teacher wellbeing can, in turn, affect the organization of educational systems as a whole (Albulescu et al., 2019). Teacher wellbeing has been found to be impacted by external factors, such as job characteristics (i.e., job resources and job demands), and by internal factors, including teachers’ emotional regulation strategies and mindset.

In recent years, various reforms and initiatives in educational sectors have been implemented to change education to better prepare the new generation for the future. Under these circumstances, changes in job characteristics have been brought along with the educational reforms. Questions as to what these educational reforms bring to teachers and how they impact teacher wellbeing are worth exploring. However, compared to the vast amount of research on general teacher wellbeing, systematic research on teacher wellbeing in educational reforms is relatively rare. Taking China as an example, the General Office of the Central Committee of China’s Communist Party and the General Office of the State Council jointly released the Opinions on Further Reducing the Burden of Homework and Off-Campus Training for Compulsory Education Students (referred to as the educational Double Reduction Policy in its shortened form) in 2021. This policy mandates a reduction in the total amount and time of commitment required by school homework and a reduction in the burden of off-campus or after-school training programs (MoE, 2021). Implementation of the policy has placed higher requirements on teachers, and their job characteristics have undergone drastic changes (Yao and Zhang, 2022). Teachers need to prolong their working hours to participate in after-class tutoring and caretaking. This demands more of their professional capabilities and requires them to play a bigger role and shoulder more responsibilities. All of these have impacted their wellbeing in various ways.

Numerous studies have explored the theoretical and practical logic of the policy itself or the changes and influences in the perspective of pupils under the policy, including their learning, psychological views (Zhang et al., 2022), and mental health problems (Wang et al., 2022). However, studies on teachers are lagging far behind other works carried out under the policy. Questions such as “What is the status quo of teacher wellbeing in China right now? How do the changes in job characteristics impact their wellbeing specifically? How do they regulate their emotions to meet new challenges? What kind of mindset is beneficial to regulate their emotions so as to preserve their wellbeing in educational reforms?” are critical but unanswered. This study aims to promote this line of inquiry.

Therefore, exploring teacher wellbeing and its links to changes in job characteristics brought about by educational reforms is of great importance and necessity. It is also conducive to understanding how teachers cope with new job characteristics and how they regulate their emotions and mindset to maintain wellbeing in a broader context. Thus, this study is of special significance as a systematic investigation into teacher wellbeing in the context of educational reforms. Furthermore, the current study is innovative because of the exploration and construction of the associations among teacher wellbeing, job characteristics, emotional regulation strategies, and mindset in a holistic approach under one unified context rather than in isolation. With this research, increased attention to and comprehensive understanding of teacher wellbeing can surely and progressively be generated.

Building upon the findings of relevant studies, the current study examined the new job characteristics that China’s Reduction Policy imposed on schoolteachers and their relationships with teacher wellbeing and teachers’ inner world (i.e., emotional regulation and mindset), highlighting the interaction among society, schools, and individuals. Based on the collected data and their findings, the current state and interaction mechanism of teacher wellbeing were systematically extracted and summarized in depth. For this purpose, the study addressed the following four research questions:

1. Has teacher wellbeing changed after the Double Reduction Policy?
2. How do the changes in job characteristics impact teacher wellbeing?
3. How do teachers’ emotional regulation strategies impact teacher wellbeing? How do they influence the relationship between teacher wellbeing and job characteristics?
4. Does mindset affect teachers’ emotional regulation and wellbeing? If so, how does this work?

## Literature and hypotheses

### Conceptualizing teacher wellbeing

There have been divergent understandings of the term wellbeing in general. Ryan and Deci (2011) defined wellbeing as optimally healthy psychological functioning and experience, which was termed as general wellbeing (Collie et al., 2015). Besides, hedonic and eudemonic perspectives of wellbeing (Ryan and Deci, 2011) were inevitably featured in the existing studies. Based on the hedonic view, wellbeing consists of pleasure and happiness, mainly measuring subjective wellbeing. This perspective focuses on three components: life satisfaction, the presence of a positive mood, and the absence of a negative mood, together often summarized as happiness (Diener et al., 1998). The eudemonic viewpoint, however, argues that wellbeing is more than just happiness but is equal to fulfilling or realizing one’s true nature, mainly focusing on psychological wellbeing, encompassing six aspects: autonomy, environmental mastery, personal growth, self-acceptance, life purpose, and positive relatedness (Ryff and Keyes, 1995). Both viewpoints regarded wellbeing as a multidimensional phenomenon (Bakker, 2015; Keeman et al., 2017), which can be reflected in the PERMA (positive emotions, engagement, relationships, meaning, and accomplishment) model put forward by Seligman (2011). In sum, “wellbeing is diverse and fluid respecting individual, family and community beliefs, values, experiences, culture, opportunities and contexts across time and change. It is something we all aim for, underpinned by positive notions, yet is unique to each of us and provides us with a sense of who we are (McCallum and Price, 2016, p. 17)”.

As a type of occupational wellbeing, the significance of teacher wellbeing has received widespread recognition by a growing number of researchers and policymakers. The term first appeared in 1998 (Scott, 1998) without a systematic definition. Varying discussions on teacher wellbeing have emerged since its initial appearance. According to Aelterman et al. (2007), teacher wellbeing was considered to be a positive emotional state resulting from a balance of environmental factors and teachers’ personal needs. Acton and Glasgow (2015) defined teacher wellbeing as a personal sense of professional satisfaction, enjoyment, purposefulness, and happiness. In general, teacher wellbeing is a multidimensional concept (Collie et al., 2015; García-Álvarez et al., 2022) encompassing various personal and contextual elements. It is a combination of positive psychological conditions and a functional balance between individual resources and challenges faced at work (Dodge et al., 2012). It is not only subjective and physiological but also objective and social, since the environmental conditions in which individuals live and work influence their perceptions and prospects for wellbeing (Benesch, 2017; Mercer, 2021). Accordingly, teacher wellbeing is diverse and fluid, taking into account individual, family, and community beliefs, values, experiences, culture, opportunities, and contexts as they change over time (McCallum et al., 2017). Therefore, to achieve and maintain wellbeing, teachers need to frequently interact with various scenes inside and outside the classroom.

According to a vast body of related works, the Organization for Economic Cooperation and Development (OECD) classified teacher wellbeing into four main dimensions: subjective wellbeing, cognitive wellbeing, physical and mental wellbeing, and social wellbeing (Viac and Fraser, 2020). Each dimension can be viewed as both an outcome and a facilitating condition related to the others, eventually affecting teachers’ overall levels of stress and future work engagement. Subjective wellbeing, also known as self-reported wellbeing, is defined as good subjective experiences that people make of their lives and people’s affective reactions to their experiences (Viac and Fraser, 2020). Cognitive wellbeing refers to how people assess their lives in general (i.e., life satisfaction) or particular life aspects (e.g., job, relationships, and health) (Luhmann, 2017). It is the series of skills and abilities that teachers require to work effectively (Horn et al., 2004), and it is concerned with how people perceive their lives as a whole or specific life facets (Luhmann, 2017). Physical and mental wellbeing is associated with health and symptoms teachers could experience (Viac and Fraser, 2020). Larson conceived social wellbeing as involving two factors: social adjustment and social support (Larson, 1993). Social wellbeing refers to the quality, strength, and depth of the social relationships around teachers, including relationships with children within the school, their parents, other professionals, and the local community (Viac and Fraser, 2020).

Previous studies have examined teacher wellbeing using different scales and approaches. However, most studies have separately investigated teacher wellbeing with one facet as the focus. Few empirical studies have examined teacher wellbeing as a unified set of the above four dimensions. For example, Mirbaha Hashemi et al. (2016) explored social wellbeing in Iran and developed a corresponding measuring tool. Ni et al. (2019) conducted a longitudinal environment-wide association study to examine physical, mental, and social wellbeing systematically and simultaneously. The present study took all four dimensions into consideration and explored teacher wellbeing comprehensively. Additionally, the teacher wellbeing section of the questionnaire was developed with the intention of filling current research gap by examining the level of teacher wellbeing in the context of the Double Reduction Policy. Therefore, one corresponding research hypothesis was formulated.

*H1*: Teachers show a lower level of wellbeing after the Double Reduction Policy.

The links between the four dimensions of teacher wellbeing and the questionnaire construction are as follows: Social wellbeing was explored by focusing on the main social interactions among teachers at work (i.e., support from society, school, colleagues, family, students, and parents of students). The job-related affective wellbeing scale (Warr, 1990) was employed to evaluate teachers’ subjective wellbeing, which is concerned with teachers’ affective reactions to their experiences at work, including positive and negative feelings. Physical and mental wellbeing was evaluated by deciding whether teachers are physically and mentally unwell, in accordance with how the OECD measures teachers’ physical and mental wellbeing (Viac and Fraser, 2020). Cognitive wellbeing was assessed by examining teachers’ professional satisfaction.

### Teacher wellbeing and job characteristics

Regarding the clarification and exploration of teacher wellbeing, its influencing factors are also worthy of concern. Numerous studies have reported factors associated with teacher wellbeing. Based on the evidence from existing studies, influencing factors can be mainly reflected in the dual aspects of material and spirituality, or, to be more specific, in the three paths of society, schools, and individuals. This can be viewed as partially corresponding to the ecological systems theory (EST) developed by Bronfenbrenner (1979). Using in-depth exploration of the interaction among society, schools, and individuals, the current study examined the new job characteristics induced by the educational reform in China and their relationships with teachers’ wellbeing and inner world.

The Double Reduction Policy in China has set new requirements and brought explicit and implicit risks for compulsory education schools and teachers in terms of after-school services, the quality of education and teaching in classrooms, and teachers’ participation (Yu and Yang, 2022). It has placed higher demands on the professional capacity of teachers; as a result, teachers are exposed to a structural shift in work intensity concerning 12 categories and 4 main factors (Qin and Li, 2022). Teachers’ non-teaching work has also increased, mainly including enhancing after-school services, reducing students’ homework burden, undergoing various checks and assessments, and completing numerous written materials (Li et al., 2022; Yang, 2022).

Therefore, the present study aimed, firstly, to investigate the effects of teachers’ new job characteristics on their wellbeing. The job demands–resources model (JD-R model) proposed by Demerouti and Bakker (2011) identifies job characteristics from two aspects, namely, job demands and job resources. Job demands refer to “the aspects of the job that require sustained physical and/or psychological (cognitive and emotional) effort or skills, which are therefore associated with certain physiological and/or psychological costs” (Bakker and Demerouti, 2017, p. 312). These demands can be either challenges (e.g., workload, time pressure, and complex tasks) or hindrances, such as role conflict and role ambiguity (Crawford et al., 2010). Job resources refer to the physical, psychological, social, or organizational aspects of the job that are “functional in achieving work goals; reduce job demands and the associated physiological and psychological costs; stimulate personal growth, learning, and development” (Bakker and Demerouti, 2017, p. 312), including opportunities for learning and development, autonomy, support, and feedback (Demerouti and Bakker, 2011; Schaufeli and Taris, 2014; Bakker and Demerouti, 2017). The JD-R model provides a systematic framework to explore teachers’ job characteristics for the present study.

This study also reviewed previous research instruments on job characteristics to lay the groundwork for the subsequently developed questionnaire. Liang (2020) conducted an empirical study to analyze how school job characteristics influence teachers’ job satisfaction based on the JD-R model using data collected in Shanghai for the Teaching and Learning International Survey (TALIS) 2018. Researchers have also paid a great deal of attention to scale development as well as classification exploration based on the JD-R model. In particular, a job characteristics scale of primary and secondary schoolteachers was developed by Wu et al. (2014) through semi-structured interviews and questionnaires. This scale, based on the reality that Chinese teachers face, identifies and analyzes the unique job characteristics of the teaching profession in China. It also summarizes the subdimensions of job demands and job resources, thus offering a useful tool for the present study to measure teachers’ job characteristics in China. New challenges brought by the Double Reduction Policy have taken up a tremendous amount of teachers’ efforts and free time. Teachers are susceptible to various personal and contextual job characteristics, which may influence their wellbeing in the long run. Thus, new dimensions were designed and added to the questionnaire, namely, the new job demands and resources after the policy. Two research hypotheses on the relationship between job characteristics and teacher wellbeing were proposed:

*H2*: Job demands in the context of the Double Reduction Policy are negatively associated with teacher wellbeing.

*H3*: Job resources in the context of the Double Reduction Policy are positively related to teacher wellbeing.

### Teacher wellbeing and emotional regulation strategies

Teaching is a highly emotionally demanding occupation (Hargreaves, 1998) and calls for a large amount of emotional labor because of its dynamic stages and complicated contexts (King, 2016; Gao and Cui, 2021). Teachers’ emotional regulation pertains to their ability to successfully regulate their emotions and appropriately interact in an educational context (Han et al., 2020). Exploring the affective aspects of teaching is crucial to pedagogical practice and teachers’ own beliefs in educational contexts (Hargreaves, 1998; Zembylas, 2004). Previous research has revealed that the antecedents and consequences of teacher wellbeing are correlated with their emotional regulation strategies (Wang et al., 2019).

Emotional regulation occurs when teachers’ emotional experiences are inconsistent with what their job requires. According to the current situations in China, Yin (2016) refined teachers’ emotional regulation strategies into three categories and seven subdimensions: surface acting (pretending and restraining), deep acting (refocusing, reframing, and separating), and genuinely expressing (releasing and outpouring). Surface acting refers to the modification of facial expressions without altering inner thoughts, while deep acting refers to an individual’s conscious adjustment of their inner feelings and experiences to express the expected emotion (Hochschild, 1983; Grandey, 2000). Genuinely expressing is the process of expressing one’s emotions authentically without changing any feelings or expressions (Yin, 2016). Emotional regulation is, therefore, a superficial to deep emotional expression progressing from surface acting to deep acting, and then to genuinely expressing (Xu et al., 2023). This study was conducted using this classification.

The usages of specific emotional strategies are shaped not only by teachers’ individual differences (i.e., social status, personality, abilities, and values) but also by teachers’ situational factors (i.e., the intensity of the teaching task, the teacher–student relationship, and emotional rules). According to the self-control theory and the conservation of resources theory (COR, one of the bases for the JD-R model), individuals have limited resources for self-control, and overconsumption of resources can diminish work effectiveness and subjective wellbeing (Hobfoll et al., 2018). Teachers confront their job demands and often exert immense physical and mental resources and efforts at work (Zhao et al., 2023). Therefore, teachers may invoke more than one emotional regulation strategy depending on the job requirements. To be more specific, when teachers are energetic and resourceful, they are more likely to employ surface acting and deep acting, both of which require different psychological resources to express the emotions required by their profession, and conversely, when individuals are exhausted of resources, they may find it more taxing to disguise or modify their emotional expressions and, hence, prefer genuine expressing. Based on the COR theory, surface acting requires more conscious engagement and energy resource consumption, so it is negatively related to wellbeing. Although deep acting also requires the expenditure of intentional effort and personal resource consumption, it can result in social resource compensation because of the cognitive adjustment of identity within the self that maintains individual satisfaction as well as positive feedback from others. Genuine expressing consumes fewer individual resources and mental energy, permitting individuals to benefit from a sense of authenticity. Thus, as a result of the interaction between environmental and individual factors, teachers’ adoption of different emotional regulation strategies will also, in the long run, have positive or negative influences on teacher wellbeing, interpersonal relationships, and achievement of educational goals at different levels (Grandey and Melloy, 2017). Moreover, emotional regulation has been proven to mediate between job characteristics and teacher wellbeing (Yin et al., 2016; Han et al., 2020).

Exploring the relationships among teacher wellbeing, job characteristics, and emotional regulation coincides with the current research trend of building systematic understanding. Thus, the current study is concerned with whether and how emotional regulation mediates the relationship between job characteristics and wellbeing under the Double Reduction Policy. In order to determine the direct and mediated relationships, the following research hypotheses were proposed:

*H4/H5/H6*: Emotional regulation strategies (surface acting/deep acting/genuinely expressing) affect teacher wellbeing.

*H7/H8/H9*: Emotional regulation strategies (surface acting/ deep acting/genuinely expressing) mediate the relationship between job characteristics and teacher wellbeing.

### Teacher wellbeing and mindset

Why do the effects of job characteristics on teacher wellbeing vary from individual to individual? Where does emotional regulation derive from? What elements can influence emotional regulation? To answer these probing questions, an increasing number of scholars argue that cognitive or psychological concepts (e.g., emotional intelligence, mindfulness, resilience, and mindset) can make a difference.

Emotional intelligence is known as the ability to effectively recognize and monitor one’s own and others’ emotions, distinguish between them, and utilize this information to guide thinking and actions (Salovey and Mayer, 1990). Emotional intelligence facilitates individuals’ maintenance of positive psychological wellbeing (Burrus et al., 2012) and contributes to higher job satisfaction by effectively dealing with workplace stress (Ouyang et al., 2015). However, recent research has questioned its positive effects and suggested that emotional intelligence has negative effects, or “dark sides” (Furnham and Rosen, 2016), which are manifested in the workplace both internally, as a detriment to wellbeing, and externally, as emotional manipulation and negative behaviors (Sun et al., 2022).

Mindfulness is often defined as a conscious state of nonjudgmental, moment-to-moment awareness arising from paying attention, on purpose, to the present moment (Kabat-Zinn, 1994; Kabat-Zinn, 2003). According to Feng et al. (2022), mindfulness plays a significant intermediary role between emotional labor and job satisfaction. However, mindfulness emphasizes the concept of “acceptance,” which means accepting the existence of negative mental experiences and coexisting with them (Kabat-Zinn, 2003), indicating that mindfulness can cause one to persuade oneself to accept a situation without making changes to the root cause or even without coming up with real solutions to the problems.

Resilience is considered as the ability to recover and “bounce back” from stressful situations (Khosla, 2017). This understanding was inspired by Ungar (2012), who described resilience as a process in which people apply personal and contextual resources to flexibly respond to varying circumstances, especially after setbacks or even failures. However, a time lag could exist in the process of adaptation between resilience and problems. Moreover, resilience cannot be perceived as a stable outcome state because it is dynamically affected by a variety of individual and situational factors (Chen, 2014; Song and Wu, 2022).

A number of psychological, social, and educational studies have also revealed the significance of mindset (Burnette et al., 2013; Heslin and Keating, 2016; Zhao et al., 2020), indicating that mindset can influence individuals’ cognitive modes and behavioral paradigms. The awakening and development of a positive mindset can bring us a new self and new achievement and success. Moreover, a sound and mature mindset is a prerequisite for being a teacher (Guo, 2017). Thus, the concept of mindset is conducive to explaining why teachers adopt different emotional regulation strategies and have different wellbeing outcomes. Mindsets embody perceptions about the adaptability of personal characteristics and mental frameworks that can influence people’s cognition, behaviors, and performance, such as intelligence and personality (Dweck, 1986; Heslin and Keating, 2016; Guo, 2017). Therefore, the present study explored mindset, instead of emotional intelligence, mindfulness, and resilience, as an underlying logic residing in individuals.

Mindsets can be categorized as a fixed mindset and a growth mindset (Dweck, 1986; Dweck, 2006). There are two distinct meaning systems between them (Hong et al., 1999). Individuals with a fixed mindset believe that the intelligence and abilities they have are fixed and cannot be changed, and that hard work cannot have an effect. They often tend to retreat from challenges and worry about how others perceive their intelligence, skills, and abilities. In contrast, people with a growth mindset are more likely to explore new possibilities and not avoid experiencing drawbacks. They always insist that they can grow, change, and succeed through hard work and effort. To determine which type of mindset teachers possess when facing the Double Reduction Policy and related job changes, the questionnaire involved the measurement of five dimensions proposed by Dweck (2006), namely, challenges, obstacles, effort, criticism, and success of others.

In general, a mindset is a particular set of beliefs, associations, and expectations that guides motivational processes (Zion and Crum, 2018). It regulates motivational processes so that both subjective and objective wellbeing outcomes are influenced. For example, previous studies have discovered the influences of mindset on blood pressure and body weight (Crum et al., 2013; Crum and Zuckerman, 2017). Thus, we hypothesized that teachers with distinct mindsets show differences in emotional regulation and wellbeing levels. The following hypotheses were proposed:

*H10*: Mindset affects teachers’ emotional regulation.

*H11*: Mindset affects teacher wellbeing.

### The present study

**Figure 1:**
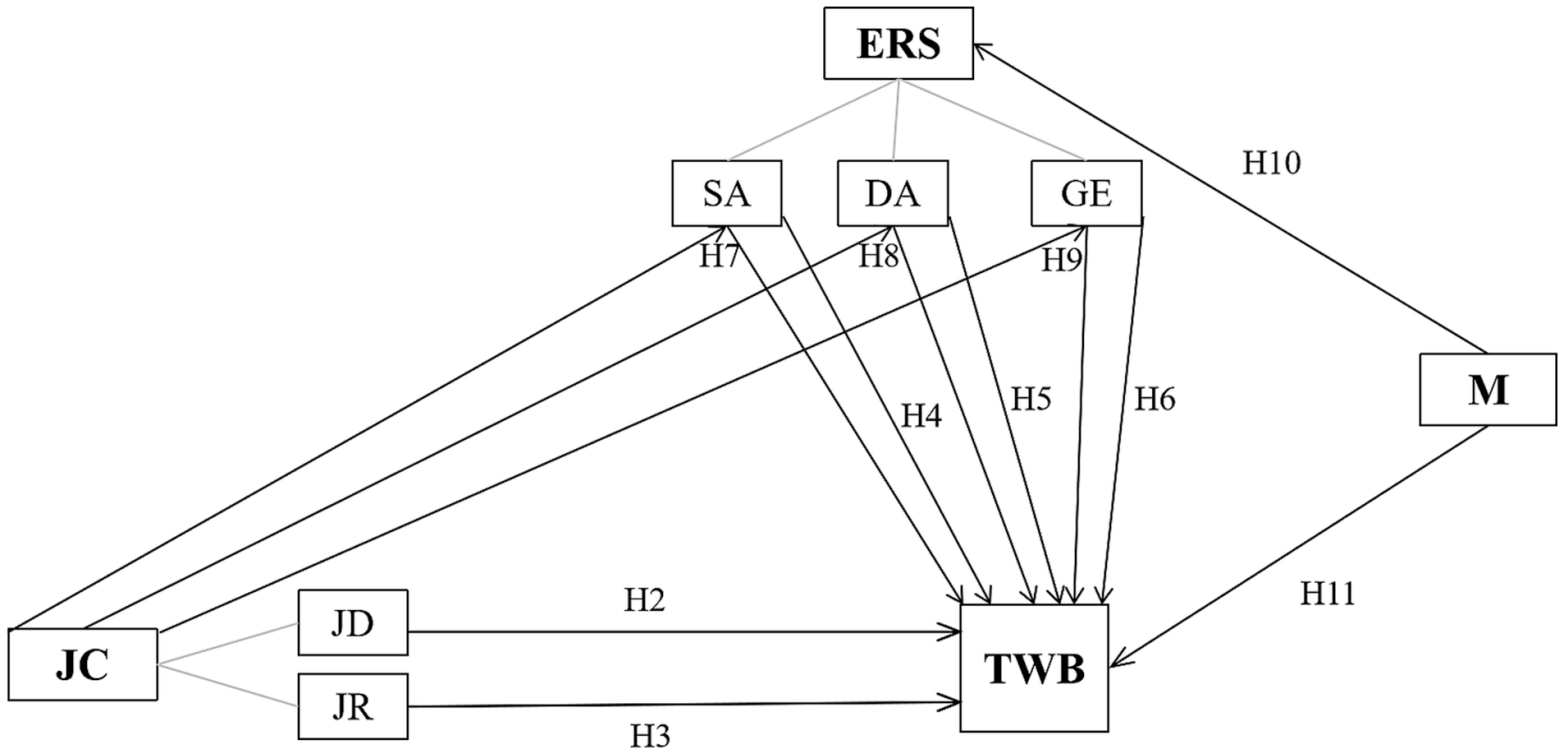
The conceptual framework of the current study. TWB, teacher wellbeing; JC, job characteristics; JD, job demands; JR, job resources; ERS, emotional regulation strategies; SA, surface acting; DA, deep acting; GE, genuinely expressing; M, mindset; arrows, causal relationships; lines, affiliation.

Permission light of the different definitions and concerns about teacher wellbeing, job characteristics, emotional regulation, and mindset, this study applied the following definition: wellbeing is not a product, but a dynamic and systematic process in which teachers regulate their emotions under complicated job characteristics to achieve harmony between physical, psychological, social, and emotional health as well as stable existence as a whole human being in the educational context. It connects to the mindset and professional development of teachers. In light of the above, the theoretical framework in Figure 1 was constructed. Hypothesis 1 is not about causal relationships, thus it is not included in the framework.

## Materials and methods

### Data collection and sample

A cross-sectional study was carried out across China, employing self-reporting questionnaires for data collection and analysis. The current study took simple random sampling and collected online questionnaire on Questionnaire Star,^1^ a professional online survey tool widely used in China. On the cover page of the online questionnaire, participants were told that the questionnaire was anonymous and the data collected would only be used for academic use. Questionnaires were designed were distributed and collected through various mobile terminals (such as WeChat, QQ, etc.), and each IP address can only be filled in once. To avoid collecting invalid questionnaires, the participants were asked to complete the whole questionnaire and was not allowed to submit before completion. 902 valid samples were finally covered. The participants were in-service teachers from cities (40.2%), towns (44.1%), and the countryside (15.6%) in China. They came from areas of eastern China (86.9%), western China (11.1%), and middle and northeast China (2%). The majority of them worked in primary schools (669, 74.2%); 89 participants worked in lower-secondary schools (9.9%), 136 in upper-secondary schools (15.1%), and 8 in integrated schools (0.9%). The sample included 70 males (7.8%) and 832 females (92.2%), with a mean age of 35 (SD = 0.941) and an average of 13.38 years in work experience (SD = 1.576). Therefore, the sample provided a diverse range of insights and perspectives at different stages of a teacher’s career and across different sectors and background trajectories.

The questionnaire only required 5 min for the teachers to answer. At the beginning, the teachers completed the sociodemographic items concerning personal, school, faculty, and occupational information. Subsequently, they provided their perceptions of job characteristics, emotional regulation, wellbeing, and mindset, in this order. As informed volunteers, all participants took part in the study anonymously and confidentially. Each participant was compensated with a digital English picture book resource package worth RMB 10,000.

### Measures

A questionnaire with four scales was employed in the present study. The scales were designed in Chinese to ensure that the participants comprehended the questions and items.

Teacher wellbeing was measured according to four dimensions: subjective, cognitive, mental and physical, and social wellbeing (Viac and Fraser, 2020). The teachers provided separate ratings according to their situations for each dimension. Cronbach’s alpha for the scale was 0.946. Social wellbeing was assessed using 6 items, covering the main social groups involved in teachers’ work (i.e., society, school, colleagues, family, students, and students’ parents). Responses were recorded on a 4-point scale ranging from 1 (never) to 4 (all of the time); higher values reflect higher frequency. Subjective wellbeing was recorded using the WARR Scale of Job-related Affective Wellbeing. This scale evaluates illbeing and wellbeing. It also distinguishes between 2 axes, 1 for anxiety–contentment and 1 for depression–enthusiasm, with each axis consisting of 6 items (i.e., tense, uneasy, worried, calm, contented, relaxed for the anxiety–contentment axis; depressed, gloomy, miserable, cheerful, enthusiastic, and optimistic for the depression–enthusiasm axis). For each item, the participants were asked how often their jobs made them feel a particular way. Responses also ranged from 1 (never) to 4 (all of the time). In terms of physical and mental wellbeing, the participants were only required to assess their overall physical condition. The options ranged from “unhealthy” to “sub-healthy” and “healthy.” The three items measuring teachers’ cognitive wellbeing were designed according to the job satisfaction scales developed by Viac and Fraser (2020) and Zheng et al. (2015).

Job characteristics were assessed with 27 items developed based on the Teachers’ Job Characteristics Scale developed by Wu et al. (2014). Four subdimensions were added to the original scale according to the research needs and the Double Reduction Policy. Job demands were measured based on seven subdimensions: workload, emotional requirements, students’ bad behaviors, entrance examination pressure, role pressure, professional ethics requirements, and new demands under the policy. Among the dimensions, the items for emotional requirements were designed according to Yin (2015). Job resources were measured based on eight subdimensions: organizational support, colleague support, parental support, hardware conditions, autonomous control, job meaning, job reward, and new job demands and resources. Responses were rated on a 4-point Likert scale ranging from 1 (never fit) to 4 (always fit). Cronbach’s alphas were 0.891 (job demands) and 0.923 (job resources).

Emotional regulation strategies were measured utilizing a 3-item scale, based on Diefendorff et al. (2005) and Yin (2012), to evaluate the extent to which teachers employ three different strategies. The participants were asked to rate each item using a 5-point Likert scale ranging from 1 (strongly disagree) to 5 (strongly agree). The higher the score on the scale, the more emotional regulation strategies were adopted. Cronbach’s alpha for the scale was −1.139, which suggests that the three strategies were inconsistent in their direction of usage. In other words, some teachers might apply all three strategies, whereas, for others, these three strategies were mutually exclusive.

To evaluate teachers’ mindsets, four items were designed according to Dweck’s (2006) classification. All items were rated on a 4-point Likert scale ranging from 1 (never fit) to 4 (always fit). A higher score indicated a preference for a growth mindset, and a lower score indicated a preference for a fixed mindset. Cronbach’s alpha for the scale was 0.922.

### Data analysis

Data analysis was performed using IBM SPSS Statistics 26.0. To test the research hypotheses, a step-by-step analysis was conducted based on the proposed conceptual framework (Figure 1). Descriptive analyses were first conducted to identify the extent of teacher wellbeing after the Double Reduction Policy was implemented and to summarize sociodemographic characteristics (age groups, marital status, teaching subjects, length of service, etc.). The mean values and standard deviations of all scales and subscales of teacher wellbeing, job characteristics, emotional regulation strategies, and mindset were examined. In addition, the variances of these domains were analyzed in depth. Independent samples *t*-tests were conducted to compare the differences between male and female genders, married and unmarried, English and non-English subjects, classroom teachers and subject teachers, and teachers with teaching posts and ordinary teachers on each dimension and subdimension of teacher wellbeing. Other potential differences among teachers from different regions, from different learning periods, teaching different subjects, and with different teaching experiences were determined using one-way ANOVA with a *post hoc* Tukey’s test. Moreover, Pearson correlation coefficients were utilized to determine the strength of association among the variables. We carried out multiple linear regression analyses to establish the statistical significance of relationships among the variables. Finally, the paths for the mediating model were examined using the PROCESS macro in SPSS 26.0.

## Results

### Measurement model

The reliability of each measurement was evaluated through Cronbach’s alpha. All measures, except emotional regulation strategies, obtained excellent alpha coefficient of more than 0.85, indicating reliability and consistency. We also performed factor analysis to test the validity of the measuring instruments. As for the exploratory factor analysis (EFA), the KMO and Bartlett’s sphericity test (KMO = 0.937) indicated an adequate sampling and satisfactory fit at *p* < 0.001. The confirmatory factor analysis (CFA) performed through Mplus Editor 8.3 suggested an acceptable factorial structure (*χ*^2^ = 1541.905, df = 278, *χ*^2^/df = 5.546, RMSEA = 0.071, CFI = 0.891, TLI = 0.873, SRMR = 0.035) in line with the conceptual framework of the research, namely, job demands, job resources, emotional regulation strategies, and teacher wellbeing, with their corresponding dimensions.

### Descriptive statistics and correlation matrix

The results of the paired samples *t*-test are shown in Table 1. The results indicated that the policy had an impact on teacher wellbeing, with a significant difference before and after the policy was implemented. Compared to the situation before the enactment of China’s Double Reduction Policy, a significant overall decline appeared in teacher wellbeing (*M* = 0.124, *t* (901) = 12.826, *p* < 0.05). The teachers demonstrated worse social, subjective, physical, and mental wellbeing than before. In sum, our first hypothesis was supported by the data analysis.

**Table 1:** Comparison of teacher wellbeing before and after the educational reform (*n* = 902).

	Before		After		MD	*t* (902)
	M	SD	M	SD		
Overall teacher wellbeing	2.66	0.413	2.54	0.458	0.124	12.826 *
Social wellbeing	2.67	0.575	2.64	0.587	0.028	2.891 *
Subjective wellbeing	2.68	0.438	2.48	0.492	0.198	14.129 *
Physical and mental wellbeing	2.21	0.509	2.03	0.553	0.181	11.533 *

*p < 0.05.

Moreover, descriptive analysis was applied to investigate and characterize the teachers’ wellbeing after the Double Reduction Policy. Based on the data analysis, the teachers showed a middle level of wellbeing (*M* = 2.54 out of 4, Std. = 0.458). Specifically, the four dimensions of teacher wellbeing were scored in descending order as follows: cognitive wellbeing (*M* = 2.71, Std. = 0.777), social wellbeing (*M* = 2.64, Std. = 0.587), subjective wellbeing (*M* = 2.48, Std. = 0.492), and physical and mental wellbeing (*M* = 2.03, Std. = 0.553). The results did not demonstrate significant differences among categories.

Furthermore, teacher wellbeing varied according to different individual variables, including marital status, educational status, school status, and teaching status. In terms of marital status, unmarried teachers experienced better physical and mental wellbeing (*t*(900) = 2.355, *p* < 0.05). In terms of academic qualification, teachers with higher academic qualifications were more inclined to use surface acting and had lower physical and mental wellbeing. Teacher wellbeing varied noticeably across regions (*F*(3, 898) = 7.324, *p* < 0.05). In particular, teachers from mid-China had relatively poorer wellbeing (MD = 2.21, std. = 0.292) than teachers from other regions. Regarding teaching subjects, English-language teachers scored significantly lower in overall wellbeing (*t*(900) = −4.072, *p* < 0.05) and in each dimension: social wellbeing (*t*(900) = −3.269, *p* < 0.05), cognitive wellbeing (*t*(900) = −2.189, *p* < 0.05), subjective wellbeing (*t*(900) = −4.193, *p* < 0.05), and physical and mental wellbeing (*t*(900) = −4.375, *p* < 0.05). Teachers with different weekly course volumes also showed significant differences in wellbeing (*F*(4, 897) = 9.139, *p* < 0.05). The fewer lessons a teacher offered each week, the better wellbeing he/she would experience. Classroom teachers reported substantially higher levels of wellbeing (*t*(900) = 2.825, *p* < 0.05), including social wellbeing (*t*(900) = 2.602, *p* < 0.05), subjective wellbeing (*t*(900) = 2.12, *p* < 0.05), and cognitive wellbeing (*t*(900) = 2.683, *p* < 0.05).

Table 2 presents the descriptive statistics of all research variables and correlation coefficients among them. For job characteristics, teachers reported experiencing slightly more job demands (*M* = 2.94, Std. = 0.574) than job resources (*M* = 2.42, Std. = 0.554). Regarding emotional regulation strategies, most teachers preferred to express emotions genuinely (*M* = 3.46, Std. = 0.914), followed by surface acting (*M* = 3.06, Std. = 1.001) and deep acting (*M* = 2.44, Std. = 0.896). On average, the teachers tended to have a growth mindset (*M* = 2.90, Std. = 0.653).

**Table 2:** Means, standard deviations, ranges, and correlation coefficients (*n* = 902).

		1	2	3	4	5	6	7
1	Overall wellbeing	1						
2	Job demands	−0.336 **	1					
3	Job resources	0.647 **	−0.052	1				
4	Genuinely expressing	0.427 **	−0.087 **	0.455 **	1			
5	Surface acting	−0.267 **	0.318 **	−0.131 **	−0.057	1		
6	Deep acting	0.069 *	−0.273 **	−0.026	−0.167 **	−0.425 **	1	
7	Mindset	0.509 **	0.131 **	0.474 **	0.320 **	−0.075 *	−0.097 **	1
	Mean	2.54	2.94	2.42	3.46	3.06	2.44	2.9
	SD	0.458	0.574	0.554	0.914	1.001	0.896	0.653
	Range	1–4	1–4	1–5	1–5	1–5	1–4	1–3.95

*p < 0.05, **p < 0.01.

Table 2 reveals the connections between teacher wellbeing and the other variables. The results indicated that job resources (*r* = 0.647, *p* < 0.01) were positively related to teacher wellbeing, while job demands (*r* = −0.336, *p* < 0.01) were negatively related to teacher wellbeing. Furthermore, surface acting (*r* = −0.267, *p* < 0.01) and deep acting (*r* = −0.069, *p* < 0.05) were negatively associated with teacher wellbeing. Conversely, genuinely expressing one’s emotions (*r* = 0.427, *p* < 0.01) showed a positive connection with teacher wellbeing. Additionally, teacher wellbeing had a significant positive relationship with having a growth mindset (*r* = 0.509, *p* < 0.01). Moreover, job characteristics were also associated with the application of emotional regulation strategies. Surface acting was positively related to job demands (*r* = 0.318, *p* < 0.01) and negatively related to job resources (*r* = −0.131, *p* < 0.01), while the correlations were reversed for the other two strategies.

### Hypothesis testing

**Figure 2:**
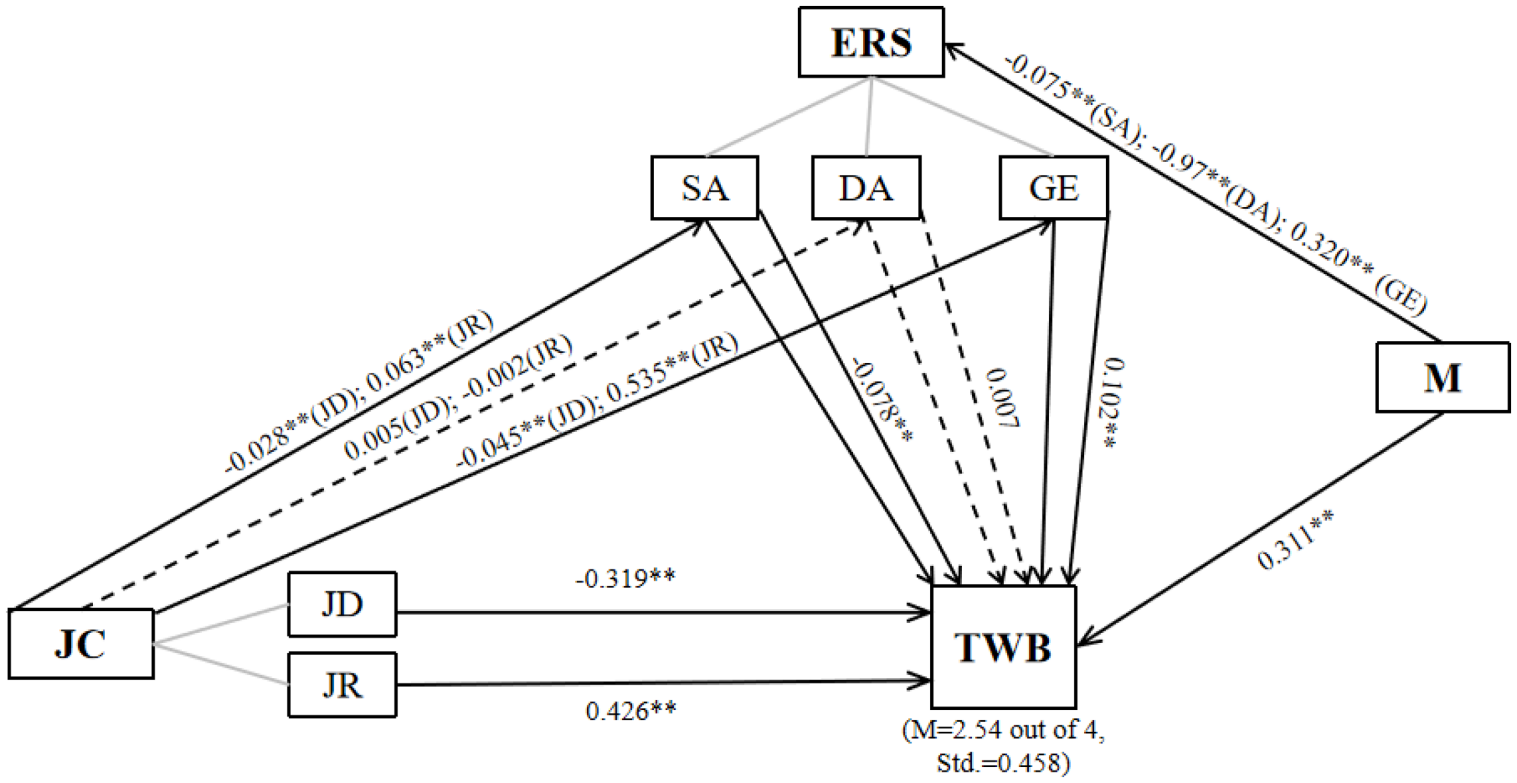
Mediation model of teacher wellbeing. TWB, teacher wellbeing; JC, job characteristics; JD, job demands; JR, job resources; ERS, emotional regulation strategies; SA, surface acting; DA, deep acting; GE, genuinely expressing; M, mindset. (**p < 0.05; solid lines, significant; dotted lines, not significant).

Multiple linear regression was conducted to determine the best linear combination of job demands, job resources, emotional regulation strategies, and mindset for predicting the wellbeing of primary and secondary schoolteachers in China after the Double Reduction Policy was implemented. The regression method of “enter” indicated that the model significantly predicted teacher wellbeing (*F*(6, 895) = 229.73, *p* < 0.05). The R-square value was 0.606, which indicated that 60.6% of the variance in teacher wellbeing was explained by the model. The beta weights, shown in Figure 2, suggested that job resources, genuinely expressing one’s emotions, and mindset contributed positively to predicting teacher wellbeing in a significant way, while job demands and surface acting contributed negatively. Deep acting did not have a significant effect on teacher wellbeing. The model indicates that job characteristics can directly influence teacher wellbeing. In addition, different usages of emotional regulation strategies have different effects on teacher wellbeing. In summary, Hypotheses 2, 3, 4, and 6 were supported, whereas Hypothesis 5 was not supported.

The standardized parameter estimates, shown in Figure 2, revealed the effect sizes and significance of the mediation model. Job characteristics had indirect effects on TWB through the path “JD → SA → TWB” (*β* = −0.028, *p* < 0.01) and “JR → SA → TWB” (*β* = 0.063, *p* < 0.01). Job characteristics also had indirect effects on TWB through the path “JD → GE → TWB” (*β* = −0.045, *p* < 0.01) and “JR → GE → TWB” (*β* = 0.535, *p* < 0.01). However, there were no significant indirect effects between job characteristics and TWB through DA, as shown in the paths “JD → DA → TWB” (*β* = 0.005, *p* > 0.01) and “JR → DA → TWB” (*β* = −0.002, *p* > 0.01). As for the underlying cause, mindset also affected teachers’ emotional regulation. Teachers with a growth mindset presented preferences for different strategies in the following descending order: genuinely expressing, deep acting, and surface acting. In summary, hypotheses 7, 9, 10, and 11 were supported, whereas hypothesis 8 was not supported.

## Discussion

### Theoretical implications

The current study confirmed our first hypothesis that teachers showed a lower level of wellbeing in educational reforms. Due to absence of systematic research on this issue, it is hard to relate the findings with previous studies. However, since the current study was conducted immediately after the educational reform, one possible explanation of this finding could be that teachers need time to adjust to drastic changes brought by educational reforms. Therefore, lowered teacher wellbeing in the early phase of educational reforms is reasonable. Teachers in China welcome the Double Reduction Policy and recognize its necessity to reduce students’ burden to bring wellbeing for a larger group of people including teachers themselves. Therefore, lowering of teacher wellbeing in educational reforms might not be ever-lasting and this depends on whether the reform is conducive and effective.

The present study also agreed with previous findings on the relationship between job characteristics and teacher wellbeing (Bakker and Demerouti, 2017; Li and Tang, 2022; Stang-Rabrig et al., 2022). As reported in the empirical study by Han et al. (2020), both job demands and job resources were significant predictors of employees’ wellbeing and performance. Stang-Rabrig et al. (2022) also proved the strong connections between job-related demands, job resources, central personal resources, and teacher occupational wellbeing under drastic social changes such as the COVID-19 pandemic. Li and Tang (2022) particularly highlighted the positive predictive effect of job resources on teacher wellbeing. Our study was in line with these related studies and further confirmed the relations (H2 and H3) in the context of the most recent educational reform in China with a reasonable sample. Job demands were negatively related to teacher wellbeing, while job resources showed a positive association. An explanation might be that teachers’ perceptions of wellbeing were contextualized by the imbalance between job demands and resources. Furthermore, this study shared the argument of Yang and Chen (2023) that until the relevant supporting facilities and systems are established, teachers have to devote considerably more time and energy to “self-dedication” as well as the completion of the transition work based on the existing heavy workload.

Although job characteristics could influence teacher wellbeing directly, the findings from this study proved that emotional regulation served as a mediator between job characteristics and teacher wellbeing. According to Han et al. (2020) and Wang et al. (2019), job demands had an indirect effect on teacher wellbeing through the mediation effect of reappraisal. Consistent with related studies, our findings partially confirmed our expectations that emotional regulation strategies would mediate the relationship between job characteristics and teacher wellbeing (H7–H9). According to a previous systematic review and meta-analytic investigation of the relationship between teachers’ emotional regulation and psychological wellbeing (Wang et al., 2019), our study also revalidated the different effects of emotion regulation strategies on teacher wellbeing (H4–H6). Echoing the previous research findings, teachers’ deep acting was not significantly relevant to their wellbeing. On the contrary, surface acting was negatively associated with teacher wellbeing, and genuinely expressing one’s emotions was related positively to teacher wellbeing.

As suggested by Lazarus and Folkman (1984), the interpretation of a stressful event is more important than the event itself. In other words, the event itself does not generate stress, and it is the way people perceive stress that is the root cause. Perceptions of wellbeing are also found to be distinctive among individuals. According to related studies, mindset reflects the adaptability of personal characteristics and mental frameworks which can influence cognition, behavior, and performance, such as personality traits and intelligence (Dweck, 1986; Heslin and Keating, 2016; Guo, 2017). In our study, mindset involves a combination of knowledge, feelings, thoughts, and actions and corresponds to teacher wellbeing. Few studies have focused on the importance of mindset; in addition, there are fewer studies on the relationship between mindset and emotional regulation or teacher wellbeing. Based on the results of our study, mindset predicted the selection of emotional regulation strategies, which significantly influenced teacher wellbeing. This supported H10–11. In terms of the exogenous influences of mindset, Mesler et al. (2021) showed that a teacher growth (or fixed) mindset is positively (or negatively) related to development of a growth mindset among students. Therefore, a focus on teachers’ mindset from an individual perspective also contributes positively to a virtuous cycle of education, which is particularly important in the context of educational reforms.

### Practical implications

Specifically, how to maintain teacher wellbeing is an ongoing practical topic. Most previous studies focused more on external measures at the school and social levels, such as establishing systems (Barbieri et al., 2019; Rahm and Heise, 2019). These studies reached a consensus that teachers’ professional wellbeing should be prioritized by educational policymakers (Mehdinezhad, 2012). This implies that more job resources are expected to meet the challenges brought by the new job demands in educational reforms. However, creating and offering job resources is a gradual process and this needs effective cooperation among the government, schools and society.

However, while changes and challenges in educational reforms are inevitable, maintaining teacher wellbeing calls for more inner force from the teachers. Wellbeing is not only the expectation that teachers can face negative events positively but also the concern for their wellbeing and how to live a better life. Recent studies have also recognized the importance of the teacher subject in educational transformation, and Cai (2019) suggested a subjective reconstruction of the teacher-self in educational transformation, including both awareness and action. Teacher wellbeing is an individual experience as well as a product of the interweaving of subjectivity and objectivity. Therefore, the self has to construct a stable identity and inner core to create events that can evoke pleasant experiences. From this perspective, teachers’ acquisition of growth and wellbeing depends on themselves. An external group or measure is only an influence to promote or a force to inspire, which does not cause the ultimate and most profound transformation. Therefore, the desire to attain a high level of wellbeing requires that teachers pay attention to their development. For in-service teachers, this is reflected in the continuous recording, research, reflection, and reconstruction of their teaching practices, which will gradually construct a systematic, complete, and profound understanding and allow them to acquire a high level of wellbeing.

Building on this foundation, this study is concerned with how to promote teacher wellbeing in terms of individual concerns and growth apart from extrinsic systems. Mindset, thus, provides us with a fresher and more powerful guide. Fostering a growth mindset and a positive self-image can help teachers have a good view of themselves holistically. Furthermore, fostering these qualities will allow teachers to combine knowledge, feelings, thoughts, and actions to respond to changes and eventually obtain a high level of wellbeing.

### Limitations and future research

Taken together, the present study advances our understanding of teacher wellbeing and offers important empirical evidence and recommendations for further research and practice. In terms of research contribution, this study collected and analyzed practical data concerning teacher wellbeing as well as how job characteristics, emotional regulation, and mindset influence teacher wellbeing. Therefore, the research findings can contribute to the richness of existing knowledge and systematic discussion of related studies. In terms of educational practice, the present study is conducive to the understanding of teacher wellbeing as well as the exploration of the underlying logic of mindset. Thus, this study inventively and systematically sheds light on how teachers can appropriately regulate their emotions and maintain wellbeing under educational reforms.

Although these results contribute to a better understanding of teacher wellbeing, some limitations and potential directions for future research also require consideration. First, this cross-sectional study was conducted under a specific context of educational reform. Thus, the results might be limited by time and context. More longitudinal studies on teacher wellbeing with a larger sample are warranted in the future, which would benefit our understanding of individual perceptions and dynamic supporting systems. Second, only online self-report measures were utilized in this study, indicating that bias might exist in the findings. This calls for future research to employ multiple sources of data to analyze teacher wellbeing, such as interviews, teacher reflection journals, and classroom observations. Finally, the current model may have overlooked other factors related to teacher wellbeing, which could be tested more extensively in future research.

In the long run, we believe that teacher wellbeing is not only an outcome but may influence emotional regulation and mindset. Therefore, with teacher wellbeing as the anchor point, teachers can shine as independent and healthy individuals who fulfill their potential. More attention to teacher wellbeing will also certainly be a step toward building a healthier educational ecology and a more harmonious society.

## Data availability statement

The raw data supporting the conclusions of this article will be made available by the authors, without undue reservation.

## Ethics statement

The studies involving human participants were reviewed and approved by Research Ethics Commitee of Capital Normal University. Written informed cosent for participation was not required for this study in accordance with the national legislation and the institutional requirements.

## Author contributions

NA: Conceptualization, Data curation, Formal analysis, Funding acquisition, Investigation, Methodology, Project administration, Resources, Supervision, Validation, Writing – original draft, Writing – review & editing. SZ: Conceptualization, Data curation, Formal analysis, Investigation, Methodology, Project administration, Resources, Validation, Visualization, Writing – original draft, Writing – review & editing. GT: Supervision, Writing – review & editing. XZ: Writing – review & editing. XK: Writing – review & editing.

## Footnotes

^1^ www.wjx.cn
