# Supplementary material for: Exploring teacher wellbeing in educational reforms: a Chinese perspective
Source: Front Psychol. 2023 Nov 10;14:1265536. doi: 10.3389/fpsyg.2023.1265536 (PMC10682978; doi:10.3389/fpsyg.2023.1265536)
Supplement: Supplementary file 1 [file Presentation_1.PDF]

Questionnaire on Primary and Secondary School Teacher Wellbeing in the Context of Double Reduction

Dear teachers:

This questionnaire survey is conducted to explore the current situation of teacher wellbeing under the Double Reduction. The academic data are strictly confidential to protect your interests and privacy, so please feel free to complete the questionnaire according to your own actual situation and first impression.

The questionnaire will take about 5 minutes to answer. After submitting, you will receive a $10,000 package of picture books. We appreciate your understanding and support!

I、Basic Information

Items	Choices				
1. Gender	Male	Female			
2. Age	20-30	31-40	41-50	51-60	
3. Educational background	High school and below	Junior college degree	Bachelor degree	Master degree	Doctor degree
4. Area	Eastern China	Mid-China	Western China	Northeast China	
5. Working region	City	Town (Including urban-rural boundary)	Countryside (Including villages and grasslands)		
6. Teaching subject	Chinese, English, Maths, History, Politics, Geography, Physics, Chemistry, Biology, Music, Art, PE, Labor Education, Comprehensiove practice, Club, After-class service				
7. Teaching experience	less than 5 years	5-10 years	11-15 years	16-20 years	more than 20 years
8. Subject teaching experience	less than 5 years	5-10 years	11-15 years	16-20 years	more than 20 years
9. Professional credential	No credential	Primary (levels 2, 3)	Intermediate (level 1)	Senior（Professor, Associate Professor）	

10. Teaching Grade	1, 2, 3, 4, 5, 6, 7, 8, 9, 10, 11, 12				
11. No. of Classes	1	2	3	4	5 and above
12. Average No. of students	less than 20	20-30	31-40	41-50	more than 50
13. Average No. of weekly course	5 and below	6-10	11-15	16-20	21 and above
14. Are your a classroom teacher?	Yes	No			
15. Do you have a management position at school?	Yes	No			
16. Marital Status	Single	Married			
17. No. of children	0	1	2	3	more than 3
18. Who takes care of your children?	myself	spouse	elders	babysitter	daycare

II、Job demands and resources

Do the following statements apply to you?

Items	A.Never	B.Sometimes	C.Usually	D.Always
19. I need to work overtime a lot.				
20. I have to spend most of my time interacting with others (e.g. students, parents and colleagues).				
21. I have to be considerate and think from the perspectives of my students and colleagues.				
22. I need to give my students a sense of security with appropriate emotions and behaviors.				
23. I need to take a lot of effort managing student disciplines.				
24. I need to put a lot of effort into motivating my students to learn.				
25. I worry about student outcomes, class rankings and promotion rates.				
26. In addition to teaching and research, I need to juggle several tasks at the same time (e.g., meetings, writing paperwork, dealing with inspections and school visits, etc.).				
27. I need to participate in competitions and public classes.				
28. I would blame myself for not taking my work seriously.				
29. With the Double Reduction, my workload has increased and my working hours have extended.				
30. With the Double Reduction, there is more student				

management work and more frequent communication needed for home-school collaboration.				
31. With the Double Reduction, I need to work harder to regulate my emotions.				
32. My leaders are able to spot my shining points.				
33. The school culture is relaxing and free, and I can utilize my expertise.				
34. My colleagues help each other and the atmosphere is harmonious.				
35. Students' parents understand and cooperate with my work.				
36. The office is well-equipped and the environment is comfortable.				
37. The school has sufficient resources for professional development (e.g., subject research, online platforms, etc.).				
38. I have autonomy to decide on my teaching and management style.				
39. I have opportunities for independent learning and development.				
40. I like my job and feel a sense of fulfillment.				
41. There is ample space for promotion in the school.				
42. The school offers various benefits (e.g., salary, allowances, holiday bonus, etc.).				
43. The Double Reduction has led to an increase in the status and remuneration of teachers.				
44. After the Double Reduction, the home-school community has a harmonious connection.				
45. Teachers receive more emotional support after the Double Reduction.				

III、Emotional regulation

Do you agree with the following statements?

Items	A. Totally disagree	B. Disagree	C. Not sure	D. Agree	E. Totally agree
46. The emotions I show at work come naturally.					
47. I need to act in order to deal with students or their parents in an appropriate way.					
48. I try to experience the emotions that I must show at work without changes.					

IV、Wellbeing

i. How often do your receive support from the following aspects?

DR=Double Reduction policy

Aspects		Frequency			
		A. Never	B. Occasionally	C. Much of the time	D. All the time
49. Support from the society	Before DR				
	After DR				
50. Support from school	Before DR				
	After DR				
51. Support from colleagues	Before DR				
	After DR				
52. Support from family	Before DR				
	After DR				
53. Support from students	Before DR				
	After DR				
54. Support from student parents	Before DR				
	After DR				

ii. How do you like your job?

Aspects	A.Never	B.Sometimes	C.Usually	D.Always
55. Teaching has always been my ideal career.				
56. I’m satisfied with what I'm doing now.				
57. If I had to choose again, I would still be a teacher.				

iii. How often do you have the following feelings?

DR=Double Reduction policy

Feelings		Frequency			
		A. Never	B. Occasionally	C. Much of the time	D. All the time
58. Tense	Before DR				
	After DR				
59. Uneasy	Before DR				
	After DR				
60. Worried	Before DR				
	After DR				
61. Calm	Before DR				
	After DR				
62. Contented	Before DR				
	After DR				
63. Relaxed	Before DR				
	After DR				
64. Depressed	Before DR				
	After DR				
65. Gloomy	Before DR				
	After DR				
66. Miserable	Before DR				

	After DR				
67. Cheerful	Before DR				
	After DR				
68. Enthusiastic	Before DR				
	After DR				
69. Optimistic	Before DR				
	After DR				

iv．How was/is your physical condition?

		**Degree**		
		A. Unhealthy	B. Sub-healthy	C. Healthy
70. Physical condition	Before DR			
	After DR			

V、Mindset

Do the following statements apply to you?

Items	A. Never	B. Sometimes	C. Usually	D. Always
71. In my work, I am not afraid of challenges and obstacles. I confront them directly.				
72. I think that hard work leads to growth.				
73. I am open to listening to others' comments and can grow from my mistakes.				
74. The success of others brings me inspiration rather than a threat.				

Here ends the questionnaire. Many thanks again for taking your valuable time to participate in this research. You will receive a picture book package worth $10,000 upon submission of the questionnaire. May you have a happy life and enjoy wellbeing!
